# Surgery

**Published:** 1858-09

**Authors:** 


### Foreign Abstracts,

CHIEFLY FRENCH, GERMAN, AND ITALIAN; OR, BI-MONTHLY NOVELTIES IN MEDICAL, SURGICAL, OBSTETRICAL, AND CHEMICAL SCIENCE. BY EMIL FISCHER, M.D.

### SURGERY.

### I. *A New Method of Amputation.* By M. MAISONNEUVE.

M. MAISONNEUVE read before the Académie des Sciences, April 26th, 1858, a note on a new operation for amputation, which he calls the *diaclastic method.* The peculiarity of this method is, that for its execution neither the knife is used for dividing the muscles, nor the saw for cutting the bones, nor permanent ligatures to arrest hemorrhage; and that, contrary to the ordinary methods, the division of the bone constitutes the first step of the operation, and precedes the division of the soft parts.

The principal object of this method is to avoid the occurrence of purulent infection, by substituting for the ordinary process of division by cutting instruments, the process of breaking, tearing, and extemporaneous ligature, the contusing action of which obliterates effectually the vascular orifices.

M. Maisonneuve uses for the execution of his method an *osteoclast*, or instrument for breaking the bone; and a powerful *serre-nœud* for the division of the soft parts. The author describes the operation in the following manner:—

"The patient having been brought under the influence of chloroform, the surgeon applies the osteoclast precisely on the spot where he intends to break the bone, taking care to protect the soft parts in contact with the instrument by thick compresses; then, giving the screw several turns, he produces the fracture; he removes the instrument, and immediately substitutes the serre-nœud, in the metallic loop of which he embraces the member ten or fifteen centimetres below the point of fracture; then, turning the screw, he gradually compresses the tissues until the circulation in the vessels is interrupted. This done, he divides the muscles to the bone by a circular incision with the bistoury, two or three centimetres below the serre-nœud, tears off by a twisting movement the extremity of the member which is attached merely by same adhering portions of muscular tissue, and finishes the operation by continuing to turn the screw of the serre-nœud until the tissues embraced in the loop of the ligature are completely divided. If the last step of the operation is conducted with prudent slowness, not a drop of blood will ooze from the wound resulting from the amputation, whatever the amputated member may be."

This singular method has been applied with success to five amputations of the leg and to one of the forearm.—(*Archives Générales*, June, 1858.)

II. *On the Scooping out of Bones as a means of preserving their Shape and Functions, and of avoiding Amputations.* By Professor Sédillot. (Read before the Académie des Sciences, March 1st, and April 12th, 1858.)

The object of this new operation is to fulfill one indication which often presents itself to the surgeon, and the great advantage of which no one contests, namely, to preserve the periosteum in order to favor the reproduction of new bone. This preservation of the periosteum has produced excellent results in the hands of Blandin, Baudens, and some other surgeons; but the dissection of the periosteum, and the extirpation of the subjacent bone, are difficult and dangerous operations. M. Sédillot proposes another process, which consists in scooping out the bone, and leaving the exterior or cortical layer intact; this layer is afterwards absorbed, and replaced by a new bone, which augments each day in volume and strength.

The operation is practiced in the following manner:—An incision is first made over the whole length of the diseased bone where it is most superficial, and farthest from the trunks of the vessels and nerves. Two other incisions, including about the fifth part of the circumference of the member, cross at a right angle the extremities of the first, and serve to form two lateral flaps. These are turned back on each side, and must contain the subjacent periosteum. The bone thus laid bare is immediately attacked by the gouge, chisel, and mallet. On penetrating the medullary canal this is dug or scooped out, made smooth, and the bone is reduced to a kind of slender shell, which is filled with charpie; the wound of the soft parts is dressed without bringing the flaps together. The advantages of this proceeding are the following:—the periosteum remains intact, and is not at all deformed; the operation is of easy execution; hemorrhage is not to be feared, the open vessels being accessible to immediate ligation; the preserved osseous surface can be cauterized or plugged according to the indications. The muscular attachments do not suffer; the wound remaining open, and retains neither plasma, nor serum, nor pus, which always find free issue. The inflammation does not go beyond the traumatic surfaces. Lymphitis, diffuse phlegmonous inflammation, pyæmia, putrid infection, are not to be feared; the wound remains simple, and heals generally without accidents.

The scooping process is applicable to suppurative hyperostosis, complicated with numerous fistulæ, which communicate with the medullary canal, to condensing and to expanding ostitis, to caries kept up by sequestra or other foreign bodies; and, according to the author's opinion, the "évidement" of the extremities of the femur, and of the tibia in severe arthritis of the knee has great advantages over the resection of this joint, and the amputation of the leg. M. Sédillot has thus far experienced neither accidents nor failure in his operation. Of ten successful cases reported by the author we give the outlines of six:—

Case V.—Coxalgia on the right side, of four years' standing, in a soldier thirty-one years of age. Luxation of the femur with caries; abscesses and numerous fistulous passages of twenty-six months' duration. Resection of the superior extremity of the femur on a level with the smaller trochanter, by separation and dissection of the periosteum. Scooping out of the osseous diaphysis to the extent of more than a decimetre. No untoward symptoms.

CASE VI.—Arthritis of the left elbow; traumatic intra-articular fractures; suppuration; grave accidents; resection of the trochlea of the humerus; scooping out of the inferior extremity of the humerus. Patient, a man thirty-one years old, is in a fair way of recovery.

CASE VII.—Caries, and numerous fistulous abscesses of the inferior extremity of the right femur. Scooping out of a portion of the diaphysis, and of the condyles. No accident. Patient, a girl thirteen years of age, is doing well.

CASE VIII.—Ostitis, caries, and necrosis of the inferior extremity of the right femur. Extraction of a large sequestrum. "Scooping." No accident. Patient, a man twenty-seven years of age, is doing well.

CASE IX.—Caries, with numerous fistulæ of the inferior extremity of the left tibia. Swelling of the tibio-tarsal articulation. Scooping out of the tibia. No accident. Patient, a girl of eighteen years, is doing well.

CASE X.—Ostitis, with abscess and fistula of the right tibia at its lower third. "Scooping." No accident. Patient, a girl thirteen years old, in fair way of recovery.—(*Gazette Hebdomadaire,* April 9th and 23d, 1858.)

III. *Observations on Hereditary Syphilis of the Bones and Cartilages.* By DR. SIGMUND.

The author communicates four cases of hereditary syphilis of the bones and cartilages. As in acquired syphilis of the bones, so also in cases of this kind the number of male patients outweighs that of the female, approaching the proportion of 2 to 1.

The disease begins to show itself in the tenth year of life. Among numerous patients, the most frequent period of commencing disease was observed between the tenth and seventeenth year. Retardation of development, leanness, pallor, frequent catarrhal and rheumatic affections which had occurred in such patients before the tenth year, are so little referable to syphilis, that they possess only small diagnostic value, as long as we do not positively know that the parents suffer from syphilis, and perhaps a comparison of the children points to the probable cause of these symptoms.

Tumefaction of the bone seems to commence without much pain, as the patients do not mention or remember anything about it, even if expressly asked; if, however, the swelling becomes more considerable, some complain of pain, which is increased with change of the weather, or sets in at night. Cutaneous ulcers accompany, very frequently, syphilis of bones and cartilages, and even often precede this disease; they are generally developed from scattered tubercles of the subcutaneous areolar tissue, as are the ulcers of the palate and throat. In three cases only the father was affected with the same form of syphilis at the time of the conception of the diseased offspring; in one case the mother. *The same form of syphilis was thus transmitted from parent to offspring, and exhibited itself in the latter only at a very long interval after birth, no other form having made its appearance during this interval.* It is remarkable, that in none of these cases abortion took place.

The treatment of these cases consisted essentially in friction with mercurial ointment, and the simultaneous use of cod-liver oil, iodide of sodium, or syr. ferri iodidi. The ulcers of the soft palate were touched frequently with nitrate of silver, and mouth-washes and gargles of crude alum were used. The cutaneous ulcers cicatrized, by the use of warm water dressings. Baths, pure air, and nourishing diet formed a part of the treatment.—(*Zeitschrift der Gesellsch. der Aerzte zu Wien*, 1858, No. 5.)

